# Tr1 Cells as a Key Regulator for Maintaining Immune Homeostasis in Transplantation

**DOI:** 10.3389/fimmu.2021.671579

**Published:** 2021-04-26

**Authors:** Yun Song, Ning Wang, Lihua Chen, Liang Fang

**Affiliations:** ^1^ Department of Immunology, The Fourth Military Medical University, Xi’an, China; ^2^ Department of Immunology, Xi’an Medical University, Xi’an, China

**Keywords:** transcription factor, biomarkers, clinical trials, transplantation, type 1 regulatory T cells, regulatory functions

## Abstract

The immune system is composed of effectors and regulators. Type 1 regulatory T (Tr1) cells are classified as a distinct subset of T cells, and they secret high levels of IL-10 but lack the expression of the forkhead box P3 (Foxp3). Tr1 cells act as key regulators in the immune network, and play a central role in maintaining immune homeostasis. The regulatory capacity of Tr1 cells depends on many mechanisms, including secretion of suppressive cytokines, cell-cell contacts, cytotoxicity and metabolic regulation. A breakdown of Tr1-cell-mediated tolerance is closely linked with the pathogenesis of various diseases. Based on this observation, Tr1-cell therapy has emerged as a successful treatment option for a number of human diseases. In this review, we describe an overview of Tr1 cell identification, functions and related molecular mechanisms. We also discuss the current protocols to induce/expand Tr1 cells *in vitro* for clinical application, and summarize the recent progress of Tr1 cells in transplantation.

## Introduction

Immunoregulation network plays an essential role in maintaining immune equilibrium of the immune system. A variety of regulatory immune cells constitute a very important and potent effector compartment of the immunoregulation network. Over the past two decades, there emerged different subpopulations of CD4^+^ T cells capable of regulating specific immune responses. As such, these cells are named regulatory T cells. The regulatory T cells are classified into the T-regulatory cells (Tregs) expressing Foxp3 (Foxp3^+^ Tregs) (1), Tr1 cells (2), Th3 cells (3), CD8^+^CD28^−^ T cells (4), human leukocyte antigen (HLA)‐G^+^CD4^+^ T cells (5) and HLA‐E‐specific CD8^+^ T cells (6). Tr1 cells have been generally characterized by their ability to secrete IL-10 and in lack of constitutive Foxp3 expression (7). Their dominant role in peripheral immune tolerance has long been considered as the primary function of Tr1 cells. In recent years, Tr1 cells have received increasing attention in medical research, particularly as the potential therapeutic tools or targets for treatment of autoimmune diseases, cancer, the prevention of organ transplant rejection, and other immune-associated disorders. In this review, we focus on the role of Tr1 cells in transplantation and their potential application.

## General Characteristics of Tr1 Cells

### Surface Biomarkers

**Figure 1:**
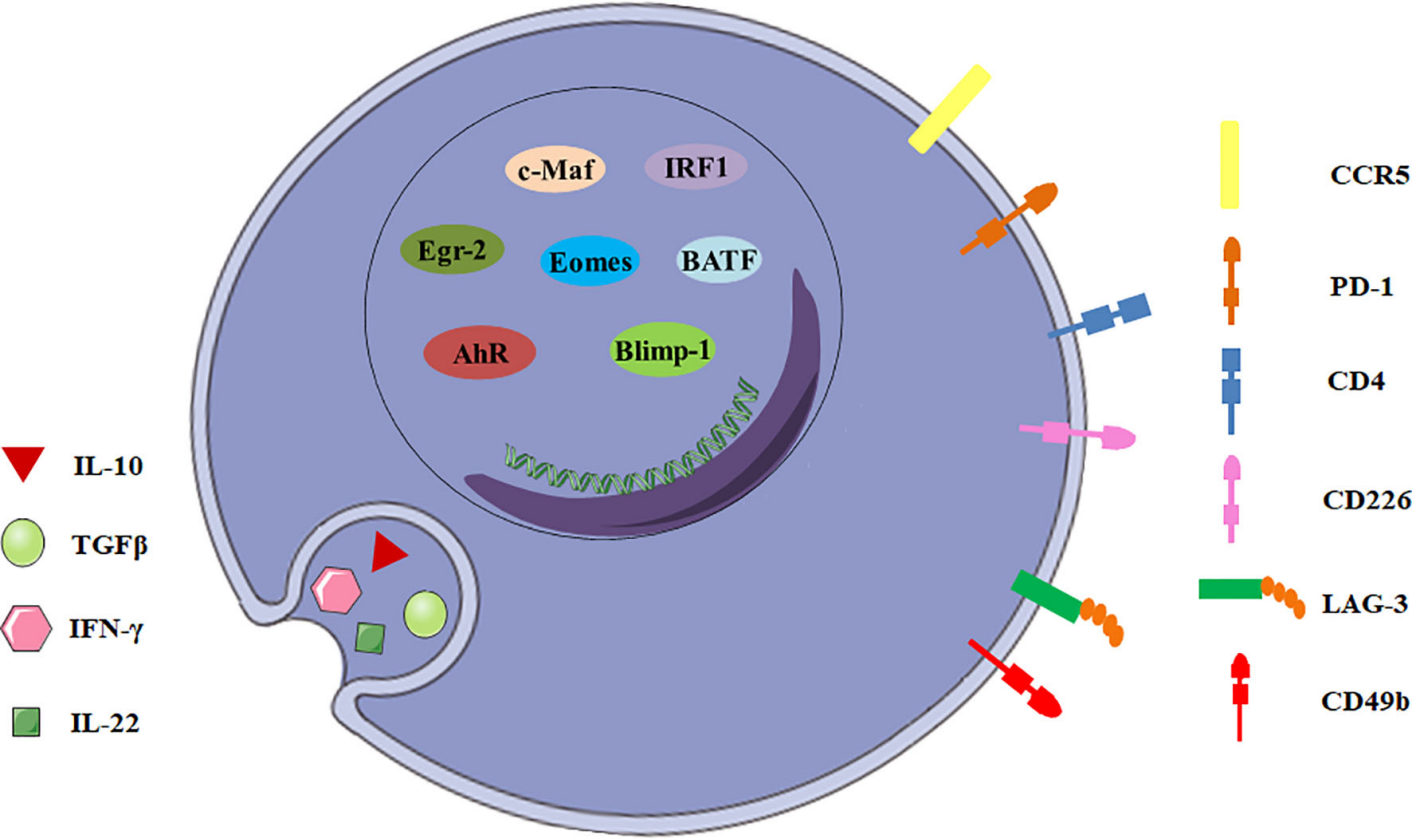
General characteristics of Tr1 cells. The biomarkers for Tr1 cells include surface molecules, secreted cytokines and transcription factors.

There are many candidate surface molecules associated with Tr1 cells, but no lineage specific biomarkers for Tr1 cells have been identified. What distinguishes Tr1 cells from other CD4^+^ T cells is the unique cytokine expression profile, IL-10^++^TGF-β^+^IFN-γ^+^IL-5^+^IL-4^-^IL-2^low/neg^ (8, 9). In 2013, a unique panel of Tr1 cell-surface markers was shown by Gagliani et al. Co-expression of CD49b and LAG-3 selectively identifies both human and murine Tr1 cells (**Figure 1**), and it can also be used to discriminate the regulatory subset from other functionally heterogeneous population of IL-10-producing CD4^+^ cells (10). CD4^+^ T cells with the CD49b^+^LAG-3^+^ phenotype secrete large amounts of IL-10 with strong IL-10 dependent suppressive activity, but low levels of IL-4, IL-17 and no Foxp3 (11). However, it was recently demonstrated co-expression of CD49b and LAG3 was not restricted to the Foxp3^-^ Tr1 cells, but was also found in Foxp3^+^ Tregs and CD8^+^ T cells following differentiation under IL-10-inducing conditions (12, 13). This finding restricts the use of LAG3/CD49b co-expression as sole markers to identify Tr1 cells.

In addition to CD49b and LAG-3, intestinal Tr1 cells co-express CCR5 and programmed cell death protein 1 (PD-1) in human and mice (**Figure 1**). CCR5^+^PD-1^+^ Tr1 cells also express IFN-γ, and could efficiently suppress T-cell proliferation *in vitro* and inhibit transfer colitis which is induced by transfer of Th17 cells in *Rag1* ^-/-^ mice (14). Moreover, Tr1 cells express some co-stimulatory molecules, including T cell immunoglobulin and mucin domain-3 (TIM-3), T-cell immunoreceptor with Ig and ITIM domains (TIGIT), cytotoxic T lymphocyte-associated antigen-4 (CTLA-4/CD152), CD226, inducible T-cell costimulator (ICOS) and other molecules, such as carcinoembryonic antigen cell adhesion molecule 1 (CEACAM-1) and class I-restricted T cell-associated molecule (CRTAM) (11, 15–17). Although none of these surface molecules can be described as surrogate markers for Tr1 cells, they might play important roles in Tr1 cells and will help to develop a new potential biomarker panel for discrimination of Tr1 cells from other CD4^+^ T cell subsets.

### Secreted Cytokines

IL-10 and TGF-β are the most important suppressive cytokines of Tr1 cells (**Figure 1**). IL-10 is an anti-inflammatory cytokine which plays an essential role in immune tolerance (18), and is first described as a key factor produced by human and murine Tr1 cells (2, 19). Although other T cell subsets can also express IL-10, IL-10 production by these cells never reaches the level of Tr1 cells (20). Tr1 cell clones display a distinct kinetics of IL-10 secretion. Tr1 cells isolated from a SCID patient transplanted with fetal liver stem cells secrete IL-10 as early as 4 hours post-activation by EBV-LCL (the host’s EBV-transformed lymphoblastoid cell line), and appear to exert their immunosuppressive functions through high expression of IL-10 (19). Human and murine Tr1 cells also secrete TGF-β, intermediate levels of IFN-γ, variable amounts of IL-5 and GM-CSF, but no IL-2, IL-4, and IL-17 (2, 21, 22). In addition, Tr1 cells isolated from healthy individuals and patients with inflammatory bowel diseases secrete IL-22, which mitigates epithelial damage and promotes barrier function of human intestinal epithelial cells (13).

### Transcription Factors

To date, a number of transcription factors are found to be important for establishing the Tr1 cell lineage, but no one is specific (**Figure 1**). Transcription factor c-Maf and ligand-activated transcription factor Aryl hydrocarbon receptor (AhR) are activated by IL-27 in human and murine Tr1 cells (15, 23). c-Maf interacts with AhR and transactivates the IL-10 and IL-21 promoters. They synergize to induce IL-27-mediated murine Tr1-cell differentiation (24). IL-6 and IL-27 utilize the same transcriptional factors and rely on IL-21 and IL-2 to induce the generation of functional murine Tr1 cells (25). However, c-Maf and AhR expressions are not exclusive for Tr1 cells. Both human and murine Th17 cells express high levels of c-Maf and AhR (24). It is still unclear if these transcription factors can be considered specific markers for Tr1 cells and master regulators for Tr1-cell function.

Furthermore, the transcription factor Egr-2 also plays a dominant role in Tr1 cells. IL-27 along with TCR stimulation induces high levels of Egr-2, which drives Blimp-1 expression in murine Tr1 cells. Egr-2-Blimp-1 axis shows a critical role in murine Tr1 cell function by promoting IL-10 secretion (26). Blimp-1 also acts in concert with the transcription factor Eomesodermin (Eomes) to activate IL-10 expression transcriptionally in murine Tr1 cells, and to repress them from differentiating into other Th-cell lineages (27). Recently, Eomes is reported to act as a lineage-defining transcription factor in human IFN-γ/IL-10 coproducing Tr1-like cells (28). In addition, Eomes is indispensable for the development of IL-10-expressing, cytotoxic Tr1 cells which control leukemia development in mice (29). Interestingly, TGF-β signaling antagonizes Blimp-1 by inducing AhR and c-Maf which shifts IL-10 regulation from a Blimp-1-dependent to a Blimp-1-independent pathway in murine Tr1 cells (30). Thus, IL-10 production in Tr1 cells might be regulated by different transcription factors in certain cytokine environments.

The other interesting transcription factors are IRF1 and BATF which are strongly induced early following TCR activation and treatment with IL-27 and are indispensable for the differentiation and function of murine Tr1 cells *in vitro* and *in vivo*. Epigenetic and transcriptional analyses reveal that IRF1 and BATF are “Tr1 epigenetic landscape” changers which influence chromatin accessibility and expression of genes required for murine Tr1-cell function (31). It has also been demonstrated that IRF4 could suppress Tr1 cell differentiation (31). However, other studies reported IRF4 could form a transcription factor complex with AhR (32), and its expression driven by ITK signaling was essential for human and murine Tr1 cell differentiation (33). Thus, the role of IRF4 in the regulation of Tr1 cells remains incompletely understood. Ultimately, most of the transcription factors discussed above have been investigated using mouse as a model organism, further studies need to be recapitulated in human cells.

## Suppressive Mechanisms of Tr1 Cells

**Figure 2:**
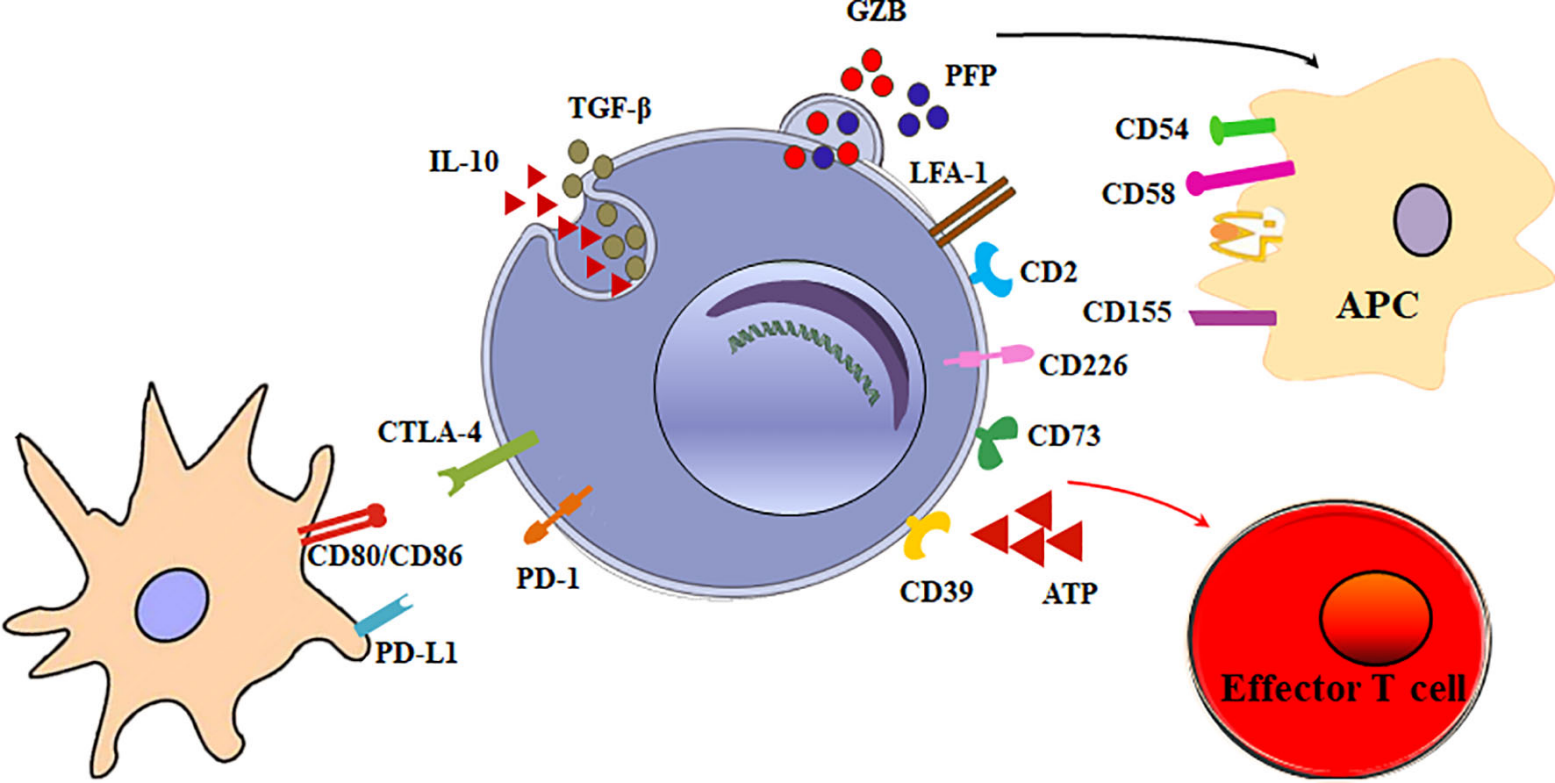
Multiple suppressive mechanisms of Tr1 cells in immune responses. The mechanisms of Tr1 cells to inhibit effector cells include: (1) Tr1 cells exert their suppressive function primarily *via* the secretion of IL-10 and TGF-β. (2) Tr1 cells contact and suppress other effector cells through inhibitory receptors, including CTLA-4 and PD-1. (3) Tr1 cells selectively kill myeloid APCs *via* secretion of Granzyme B and perforin to inhibit effector T cells. (4) CD39 and CD73 on Tr1 cells produce adenosine which increase intracellular cAMP levels and disrupts effector T cell metabolism.

The main function of Tr1 cells is to suppress effector cells. The mechanisms of Tr1 cells to inhibit effector cells are as follows (**Figure 2**).

### Immunosuppressive Cytokines

Although multiple suppressive mechanisms of Tr1 cells have been identified, the main mechanism is cytokine-mediated. Tr1 cells exert their suppressive function primarily *via* the secretion of IL-10 and TGF-β. IL-10 limits the immune responses by modulating the function of T cells, antigen-presenting cells (APC), and B cells. IL-10 and TGF-β inhibits the function of murine effector T cells by suppressing IL-2 and IFN-γ (34). IL-10 also downregulates the expression of co-stimulatory molecules, MHC class II molecules and proinflammatory cytokines by APC (9, 35). In addition, IL-10 can promote human B cell isotype switching (36). TGF-β mediates the immunosuppressive functions of human Tr1 cells partially by inhibiting cytokine production in T cells and differentiation of murine Th1 and Th2 cells (37).

### Cell-Cell Contacts

Tr1 cells exert their immunomodulatory role mainly through IL-10 and TGF-β production. Nevertheless, a mechanism implicating direct cell-cell communication has also been involved. When activated *via* stimulation of T-cell receptor (TCR), Tr1 cells can express inhibitory receptors, including cytotoxic T lymphocyte antigen 4 (CTLA-4) and programmed death-1 (PD-1). Blocking of CTLA-4 or PD-1 could inhibit the suppressor activity of human Tr1 cells (16). This contact suppression mechanism is currently unclear. It is reported Foxp3^+^ Tregs also express CTLA-4 and PD-1. Treg PD-1 and dendritic cell (DC) PD-L1 interaction or Treg CTLA-4 and DC CD80/86 interaction could induce tolerogenic dendritic cells or make the DCs unable to present a specific antigen. Further research is necessary to determine whether these inhibitory receptors on Tr1 cells exert the inhibitory effect in the similar mechanisms.

### Cytotoxicity

Granzyme B and perforin cytolysis is another important suppression mechanism of Tr1 cells. LFA-1, CD2, and CD226 are expressed at high levels on human Tr1 cells, while the myeloid APCs express the ligands of these molecules, CD54, CD58, CD155, and CD112. The receptors/ligands direct interaction is critically involved in the specific killing of myeloid APCs by Tr1 cells (38). The selective killing of myeloid cells results in inhibition of all effector T cells, and represents an additional modulatory mechanism of Tr1 cells. Furthermore, it is found that IL-10-induced Tr1 cells are able to kill myeloid leukemia in a human leukocyte antigen (HLA) class I-dependent but antigen-independent manner (39).

### Metabolic Disruption

Tr1 cells have developed specific metabolic programs to support their differentiation and suppress effector T cells. Human Tr1 cells generated in the presence of tumor cells express ectonucleotidases (CD39 and CD73) and Cyclooxygenase 2 (COX-2). Adenosine, one of the major immunosuppressive factors is produced by CD39 and CD73, and prostaglandin E_2_ (PGE_2_) is the product of COX-2. They bind their receptor A2aR and EP2R respectively, and increase intracellular cyclic adenosine monophosphate (cAMP) levels *via* the enzymatic hydrolysis of extracellular adenosine triphosphate (eATP), a strong pro-inflammatory signal. Elevated cAMP disrupts effector T cell metabolism (40). In addition, AhR boosts Tr1 cell differentiation through HIF1-α degradation, while eATP and HIF1-α induced by inflammation destabilize AhR and inhibit Tr1 cell differentiation. Crosstalk between HIF1-α and AhR regulates the differentiation of Tr1 cells through a metabolic program associated with aerobic glycolysis (41).

Taken together, Tr1 cells promote immunological tolerance through multiple mechanisms. It is tempting to reason the suppressive mechanism of Tr1 cells in patients might be associated with the origin of Tr1 cells, the stage of the disease, and pathological microenvironment.

## Tr1 Cell Induction

The induction of Tr1 cells were first found in the periphery upon antigen exposure under tolerogenic conditions (42). Until now, several methods have been established to induce Tr1 cells *in vitro* and *in vivo* which are essential for studying the role of Tr1 cells in transplantation and for developing appropriate therapeutics. Tr1-cell inducers include:

### Cytokines

**Figure 3:**
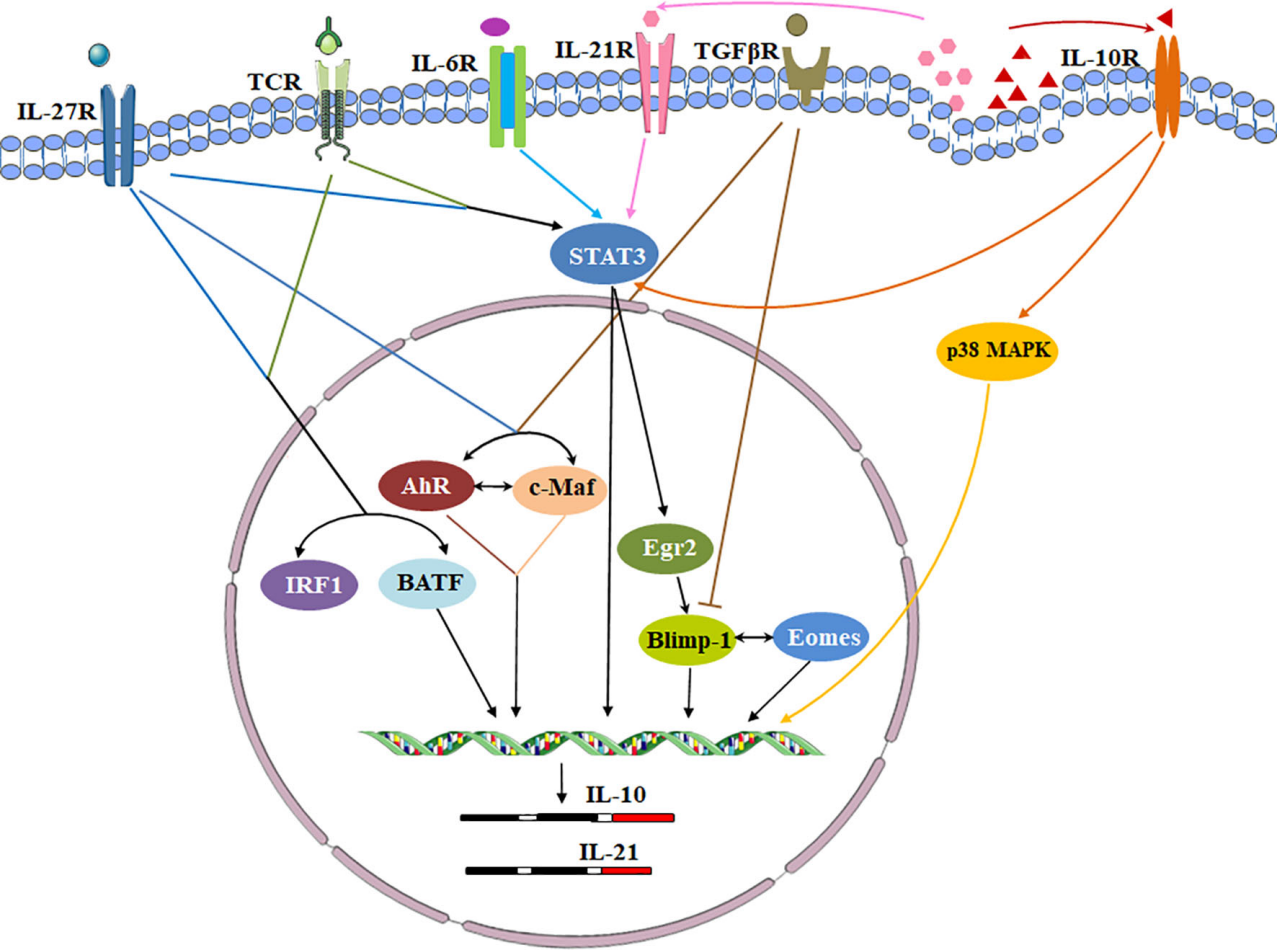
Transcriptional regulation of signaling pathways during Tr1 cell induction. Cytokines, cytokine receptors and transcription factors establish a signaling transduction network to induce Tr1 cells *in vitro* and *in vivo*. IL-27 is a key cytokine in these pathways. IL-27 activates c-Maf and AhR. IL-27 along with TCR stimulation also induces high levels of Egr-2 which drives Blimp-1 expression. Conversely, TGF-β signaling antagonizes Blimp-1 by inducing AhR and c-Maf. c-Maf, AhR and Egr-2-Blimp-1 axis promote IL-10 and IL-21 secretion. The autocrine IL-10 and IL-21 maintain Tr1 cell stability *via* p38 MAPK and STAT3 respectively. Eomes is another key transcription factor. Eomes is dependent on IL-27, and interacts Blimp-1 to activate IL-10 expression. IRF1 and BATF which induced by TCR activation and IL-27 prepare the chromatin landscape for induction of the Tr1 gene network. IL-6 and other STAT3 activating cytokines might utilize the same transcriptional factors to generate Tr1 cells.

Cytokines, cytokine receptors and transcription factors establish a signaling transduction network to induce Tr1 cells *in vitro* and *in vivo* (**Figure 3**). IL-10 and other cytokines are used for Tr1 cell generation. Human and murine Tr1 cells are induced by chronic stimulation of CD4^+^ T cells with IL-10 (2). Several methods have been identified to induce murine Tr1 cells. STAT3-dependent activation by the IL-10/IL-10R interaction is the main driving force, and other STAT3 activating cytokines such as IFN-α, IL-6 and IL-27 also promote murine IL-10-producing Tr1 cells (25, 43–45). Among them, IL-27 is the best-studied cytokine. However, the role of IL-27 in inducing human Tr1 cells remains unclear. Moreover, IL-10 in combination with anti-CD45RB mAb and Rapamycin or G-CSF is good for induction of murine Tr1 cells *in vivo (* 46).

The interesting alternative ways to generate human Tr1 cells are to constitutively over-express IL-10. The use of lentiviral vector (LV)-mediated IL-10 gene transfer converts human conventional CD4^+^ T cells into Tr1-like cells. The method allows the generation of a large number of Tr1 from the peripheral blood of patients for clinical practice (39, 47). In addition, APVO210, a bispecific fusion protein composed of an anti-CD86 antibody fused with monomeric IL-10 is used for inducing human Tr1 cells *in vivo* which had a lesser pleiotropic compared to IL-10. APVO210 differentiates CD14^+^ monocytes into tolerogenic DCs. The tolerogenic DCs are able to induce Tr1 cells by producing high levels of IL-10 (48). However, the new clinic data show the repeated doses of APVO210 could induce increasing titers of anti-drug antibodies (ADA) which lead to the failure of the clinical trial. Further improvements of APVO210 to reduce the ADA reactions include replacement of the critical linker and a reduction in mutations in other areas of the platform sequence.

### Signaling *via* Surface Molecules

Additional stimuli are involved in the differentiation of Tr1 cells *via* some surface molecules. Costimulation of TCR with CD2 specifically induces the differentiation of human Tr1 cells. Costimulatory receptors are also involved in Tr1 cell differentiation. IL-27 induces costimulatory receptor ICOS expression on murine Tr1 cells. ICOS/ICOS ligand interaction induces c-Maf expression that may further enhance stable IL-21 production. ICOS, c-Maf, and IL-21 coordinately work together to promote differentiation of murine Tr1 cells (15). In addition, the glucocorticoid-induced TNFR-related protein (GITR)/GITR ligand (GITRL) interaction promotes the differentiation of murine Tr1 cells (49). Interestingly, the complement regulatory protein CD46 and CD55 costimulations could facilitate the differentiation of human Tr1 cells. CD46-costimulated human T cells switch from Th1 cells to Tr1 cells (50, 51). CD55 costimulation could drive human Tr1 cell activation, expansion, and function (52). These data would suggest that some surface molecules emerge as key regulators of Tr1 cells.

### Dendritic Cells and Other Cells

Dendritic cells are the major inducers of Tr1 cells. Tr1 cells are generated by co-culture of naïve CD4^+^T cells and dendritic cells. This generation depends on the production of IL-10 by DCs. Human Tr1 cells can be induced by repetitive stimulation of human naive CD4^+^ T cells with allogeneic immature monocyte‐derived DCs (7). Furthermore, human tolerogenic DCs, termed DC-10 are currently the best Tr1-cell inducer *in vitro*. DC-10 are induced from human peripheral blood CD14^+^ cells by the addition of GM-CSF, IL-4 and IL-10 for 7 days. DC-10 are able to secrete high levels of IL‐10, and strongly express the signaling tolerogenic molecules ILT4 and HLA‐G. ILT4/HLA‐G interaction and signaling enhance autocrine IL‐10 production by DCs and contribute to T‐cell anergy and the induction of Tr1 cells (53, 54).

In addition, Murine CD5^+^ B-1a cells induce naive CD4^+^ T cells into Tr1 cells through an IL-10-independent pathway. CD86-mediated cell-cell interactions are essential for the induction of Tr1-of-B1a cells (55).

### Microbiota

Microbiota have been shown to modulate Tr1 cell differentiation, and are gaining more attention recently. It has been reported nonpathogenic bacterium Vitreoscilla filiformis lysate (Vf) induces tolerogenic DCs by TLR2 and drives the differentiation of murine Tr1 cells. Topical treatment with Vf significantly reduces atopic dermatitis-like inflammation in NC/Nga mice (56). Interestingly, another bacterium derived from food Lactobacillus pentosus, KF340 (LP340) modulates the DCs in a TLR independent fashion to generate murine Tr1 cells from CD4^+^T cells (57). Probiotic bifidobacterium breve activates intestinal CD103^+^DCs to produce IL-10 and IL-27 *via* the TLR2/MyD88 pathway thereby inducing murine Tr1 cells in the large intestine. Oral bifidobacterium breve administration could ameliorate murine intestinal inflammation through the induction of intestinal Tr1 cells (58). In addition, microbiota-derived short-chain fatty acids (SCFAs) promote murine Tr1 cell induction in Blimp-1-dependent manner, and oral feeding SCFAs inhibits murine colitis induced by dextran sulfate sodium (DSS) (59). These findings demonstrate that intestinal microenvironment is crucial in Tr1-cell differentiation.

### Others

AhR ligands TCDD and FICZ induce human Tr1 cells through interactions mediated by AhR and c-Maf (24). Human and murine Tr1 cells are also induced in the presence of immunosuppressive drugs, vitamin D3 and dexamethasone (60). In addition, ECM components hyaluronan (HA) promote the induction of human and murine Tr1 cells by CD44 cross-linking and signaling through p38 and ERK1/2 (61). Moreover, the anthelmintic drug, praziquantel and a soybean-derived serine protease inhibitor, Bowman-Birk inhibitor (BBI) can induce human and murine IL-10-producing Tr1 cells *in vitro*, respectively (62, 63). These recent advances will help in developing new protocols for Tr1 cell induction and broaden their potential application.

Besides, there are some negative regulators of Tr1 cell differentiation. Metallothioneins inhibit STAT1 and STAT3 phosphorylation, thereby impairing IL-10 expression and preventing IL-27-induced murine Tr1 cell development *in vitro* and *in vivo (* 64). The oxysterols, oxidized forms of cholesterol also inhibit IL-27-induced murine Tr1 cell function *via* liver X receptor signaling (65).

Taken together, Tr1 cells are mainly induced peripherally. A variety of extrinsic factors could promote Tr1 cell differentiation. However, the immune microenvironment is full of complicated exogenous components, and therefore could hardly present only inducing signals to Tr1 cells. Direct targeting endogenous components, such as epigenetic factors, transcription factors, and metabolism-related molecules might be a promising approach to induce Tr1 cells *in vitro* and *in vivo*.

## Role of Tr1 Cells in Transplantation

Tr1 cells exert a pivotal immunosuppressive role, and associated with transplantation tolerance. In human renal transplantation, the number of Tr1 cells are correlated with stable graft function positively (66). Perioperative infusion of donor bone marrow cells (DBMC) could increase the frequency of Tr1 cells and enhance the long-term transplantation tolerance in kidney transplant recipients (67). In addition, Tr1 cells promote graft tolerance in the mouse models of pancreatic islet transplantation through induction of host Tr1 cells, and the therapeutic efficacy is strictly dependent on the antigen specificity of Tr1 cells (68, 69). Recently, the immune regulatory role of Tr1 cells has been further confirmed in the islet allografts of rhesus macaques. Apoptotic donor leukocyte infusions could improve long-term allograft survival which is associated with significant increased Tr1 cells (70).

Graft-versus-host disease (GVHD) is an immunological complication commonly observed after allogeneic hematopoietic stem cell transplantation (HSCT). There are a high proportion of Tr1 cells in severe combined immune-deficient (SCID) patients who have received HSCT. These Tr1 cells of donor origin are specific for host HLA-Antigens and induce tolerance in the recipients (19). In patients with high-risk/advanced stage hematologic malignancies undergoing T-cell depleted (TCD) haploidentical-HSCT (haplo-HSCT), the IL-10-induced alloantigen specific donor Tr1 cells could inhibit the donor-vs.-host-reactivity. In addition, β-thalassemia is a genetic disease and can be cured with HSCT. Tr1 cells in transplanted patients are observed at high frequencies, and they are playing a central role in sustaining persistent mixed chimerism (PMC) and maintaining long-term tolerance after HSCT (71, 72). Furthermore, in murine utero hematopoietic cell transplantation (IUHCT) models which has the potential to cure congenital hematologic disorders, Tr1 cells are increased in the spleen and bone marrow after early-gestation IUHCT and may contribute to reciprocal tolerance (73).

Great efforts have been made in understanding the mechanisms of Tr1 cell differentiation after HSCT. It is found that the transcription factor Eomes promotes the generation of Tr1 cells during murine bone marrow transplantation. Eomes acts with Blimp-1, and its expression requires T-bet and donor macrophage-derived IL-27 (27). These findings emphasize the immunomodulatory role of Tr1 cells in GVHD.

## Clinical Applications in Transplantation

### Approaches to Generate Tr1 Cells for Therapeutic Use

Tr1-cell therapy has become the focus of many of studies, but the differentiation and expansion capacity of Tr1 cells limits its clinical use. Until now there are several good manufacturing practice (GMP) compatible protocols to expand human Tr1 cells *in vitro* which allows Tr1 cells as a therapeutic product using in clinical applications. (1) T10 cells are Tr1-cell enriched product, and can be used in kidney transplantation and other immune-mediated diseases. Irradiated donor-derived DC-10 cells are co-cultured with host CD4^+^ T cells and exogenous IL-10 for 10 days to generate T10 cells. This product is donor-specific and can promote/restore Ag-specific tolerance (74). (2) Donor PBMC (responder cells) and host CD3-depleted cells or DCs (stimulators) are co-cultured for 10 days in the presence of IL-10. IL-10-anergized donor T cells (IL-10-DLI) are obtained after the mixed lymphocyte reaction (MLR). IL-10-DLI contain Tr1 cells, and have a phenotype similar to untreated donor PBMC. These Tr1 cells have significantly improved immune reconstitution after haplo-HSCT without increasing the risk of GVHD (75, 76). (3) An improved protocol for *in vitro* differentiation of human Tr1 cells, termed T-allo10 is established. T-allo10 cells are generated from donor-derived CD4^+^ T cells stimulated with host-derived DC-10, in the presence of IL-10, and contain up to 15% of CD49b^+^LAG-3^+^ Tr1 cells (9).

### Clinical Trials

At present, Tr1-cell therapy has been demonstrated to be safe and effective in some clinical trials. In a “The ONE Study” trial (an integrated European Union-funded project to kidney transplantation, NCT01656135), a clinical-grade compatible protocol is developed to produce donor-specific Tr1 cells (T10 cells) *in vitro*. It was also found in the Reference Group Trial that the tolerogenic gene signature of the Tr1 cells remains stable in kidney transplant patients receiving immunosuppressive treatment (74). In addition, there is a single center, non-randomized prospective phase I–II clinical trial (ALT-TEN protocol, IS/11/6172/8309/8391). Patients affected with high-risk/advanced stage hematologic malignancies are treated with haplo-HSCT and Tr1-cell therapy at the same time. The cellular therapy improves the immune reconstitution and prevents relapse and GVHD in HSCT transplanted patients (76). Another new Phase I trial (NCT03198234) using T-allo10 cells was initiated to prevent GvHD after mismatched related or unrelated unmanipulated HSCT in hematologic malignancies. The first results were safe and promising, but further clinical effects are still under investigation (9).

## Future Challenges

While it is well established that Tr1-cell therapy may be of therapeutic value for transplantation, many questions are needed to be resolved. (1) The medicinal production contains Tr1 cells and other regulatory/effector cells. It is needed to isolate Tr1 cells from the cell mixture. The biomarkers LAG-3 and CD49b for Tr1 cells facilitates the identification of target cells, but co-expression of CD49b and LAG3 is also observed in Foxp3^+^Tregs and some CD8^+^T cells (12). Ideal surface markers for Tr1 cells still remains to be determined. (2) The expansion capacity of current protocols is limited. Some new approach can be applied to generate Tr1 cell *in vitro* and *in vivo*, such as the use of bidirectional lentiviral vector (LV) encoding for human IL-10 and Δ nerve growth factor receptor (NGFR) (39), and the application of APVO210, a bispecific fusion protein (48). (3) Although the Tr1 cell therapy is demonstrated effective and safe in some completed clinical trials, the regulatory and transcriptional mechanisms of Tr1 cells remain unclear. It requires further understanding of their biological features to improve the stability and safety of Tr1 cell medicinal product. (4) Until now, Tr1 cells are well defined. However, whether Tr1 cells are a heterogeneous cell population which contains several subsets with distinct origins, locations, markers, and functional properties is debatable. Distinct Tr1 cell subsets might use different suppressive mechanisms to regulate immune responses. For instance, in the intestines, human and murine Tr1 cells specifically co-express CCR5 and PD-1, and produce high amounts of IFN-γ. Moreover, LAG3 is expressed on the cell surface only after T-cell receptor stimulation. These findings suggest a possible heterogeneity among Tr1 cells. Now, technological advances have allowed to gain new insights into the heterogeneity, surface markers, and signaling mechanisms of Tr1 cells. Single-cell transcriptome analysis of Tr1 cells in various lymphoid and non-lymphoid tissues have enabled researchers to study the Tr1 cells in detail, and deepen our understanding of the phenotypic and functional heterogeneity of Tr1 cells.

## Conclusion

In the past twenty years, the knowledge of Tr1 cells is growing due to the significant achievements in understanding the function of Tr1 cells. The discovery of specific biomarkers and optimized clinical grade protocols to generate Tr1 cells promote Tr1 cell-based therapy in transplantation and many diseases. However, as a key modulator in the immunoregulation network, further investigations are required to address the molecular mechanisms controlling Tr1-cell differentiation and function. The recent studies find some transcription factors that contributed to differentiation and metabolism of Tr1 cells, but none of them is lineage specific. In addition, the current strategy to produce a large number of Tr1 cells *in vitro* broadens Tr1-cell based therapy, but long-term researches are needed to prove the safety and efficacy of the clinical application. The next generation Tr1 cell, such as chimeric antigen receptor (CAR)-Tr1 cells may show new insights into cellular therapy. Future studies in Tr1 cells should be directed toward promoting or restoring the balance of immune system in transplantation and other immune-mediated diseases.

## Author Contributions

LF, YS, and LC wrote the manuscript. NW helped in writing the manuscript. All authors contributed to the article and approved the submitted version.

## Funding

This work was supported by the National Natural Science Foundation of China (81872315, 82071848 and 81571531) and Natural Science Basic Research Plan in Shaanxi Province of China (Program No. 2021JQ-778).

## Conflict of Interest

The authors declare that the research was conducted in the absence of any commercial or financial relationships that could be construed as a potential conflict of interest.
